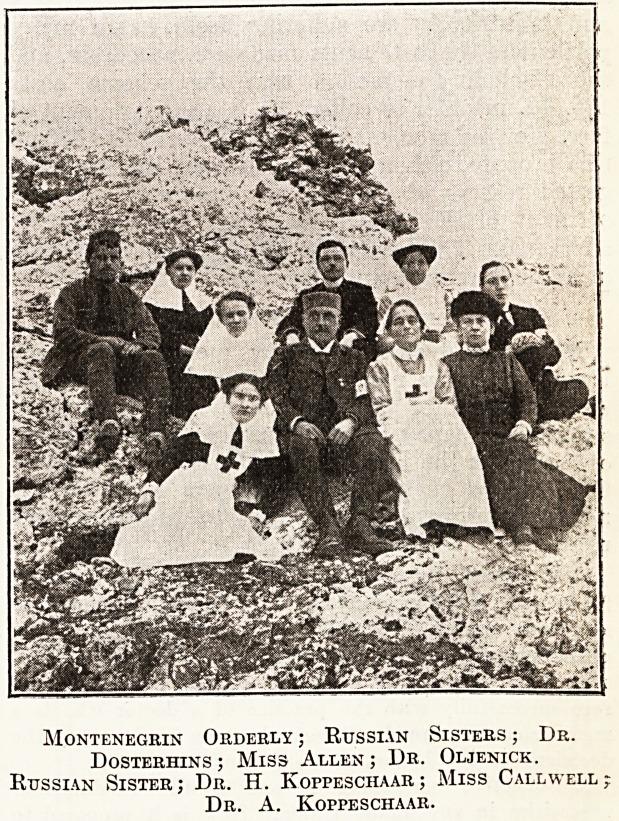# British Workers in Foreign Countries

**Published:** 1913-08-23

**Authors:** 


### BRITISH WORKERS in FOREIGN COUNTRIES.

### WITH THE WOUNDED IN MONTENEGRO—I.

The Hospital circulates not only within the British Empire, but has regular subscribers in many foreign countries, not only in Europe and the United States, but even further afield. Those members of our race who are working overseas, or in continental countries, or in the United States, or in India, or in any British dependency beyond the seas, naturally have experiences which should prove instructive and intensely interesting to our readers. We have, therefore, decided, in compliance with the general wish, to publish periodically contributions from workers abroad, dealing with their experiences in a practical and instructive way. We hope our readers will accept this information, and that those of them who have correspondents and friends working outside the British Islands will inform their friends of the establishment of this new department in The Hospital, and so make it speedily known, and, as we hope, useful to such friends and indirectly to themselves. Those of our readers who care to send us the names and addresses of British workers resident abroad will co-operate in a way we shall greatly appreciate.

### War News at Vienna.

We have pleasure in commencing the Series by publishing the following account of the experiences of Miss Emily Callwell, a trained nurse, who went out independently to the Balkans and did good service by nursing successively Turks, Bulgarians, and Montenegrins.

I was in Vienna when the news of the desperate fighting around Scutari in the first week of February induced two friends and myself to pack up a few necessaries and hurry to Trieste, where we embarked for Cattaro. This is a Dalmatian port lying at the head of an inlet of the Adriatic sea, the luxuriant vegetation, orange trees and vines, roses and carnations making a wonderful contrast to the Land of the Black Mountain towering above. A drive of eight hours up a wonderfully constructed road over the pass 3,000 feet above the sea brought us to Cettinje, surrounded by frozen peaks shining brightly in the moonlight. Here work in abundance was waiting for us.

### Hospitals.

There was practically no organisation at the front; after the long armistice most of the foreign doctors had left, and thousands of sick and wounded were brought by steamer and road to Podgoritsa and Cettinje, where small preparation had been made to receive them. Money poured in from sympathisers abroad, but with the best will in the world it could not be immediately utilised in even providing beds as there were few in the country and weeks elapsed before enough were made. I shall never forget my first sight of the great Parliament Hall with scores of men lying in three rows on the polished floor on straw mattresses crowded together. The brilliant electric light and the walls covered with gaudy paintings made the incongruity of grimy wounded soldiers there the more apparent. All the public buildings were quickly filled, even the theatre and the little kindergarten school, and as more and more arrived by motor ambulances they were billeted in private houses through the little town of 4,000 inhabitants until there were 3,000 to be looked after. Montenegro possesses but one hospital of sixty beds or so, thoroughly up-to-date as it has only been built two or three years, with a first-rate surgeon and trained Russian sisters, but a large temporary hospital was established in the barracks, and Bohemian surgeons did good work here throughout the war. A Dutch doctor also dealt with great numbers of wounded in the Gymnasium, formerly the King's palace, and he was fortunate in having a Russian sister to help him who had been through the Russo-Japanese War, and he was later joined by his wife, also a doctor.

### Food.

In one way the Gymnasium's beneficent activities spread over a wider area than its own walls, for in a ramshackle old building in the yard cooking was done for at one time as many as six hundred, there being no kitchens in the huge Government building where so many patients were, nor in the schoolhouses which had been taken for medical cases. And very well done the cooking was; most of the soldiers had never had such abundant diet in their own poor homes. Bread and meat are their staple food, and the fat making a thick coating on the soup is much appreciated.

Montenegrin Orderly; Russian Sisters; Dr. Dosterhins; Miss Allen; Dr. Oljenick. Russian Sister; Dr. H. Koppeschaar; Miss Callwell; Dr. A. Koppeschaar.

### The Sour Milk Habit.

Cabbage, beans or potatoes, macaroni or rice, and plenty of onions and seasoning made variety, and soup was served for both dinner and supper, accompanied by the boiled meat and large lumps of good brown bread. For breakfast there was tea, very weak and very sweet and without milk, as drunk in the East. For those unable to take the ordinary diet milk was ordered, but enough could not always be provided, and I had often to eke out the supply by using artificial brands. These were greatly disliked by the patients, and new milk nearly as much so; they are accustomed to drink it always sour, and friends used to bring them supplies in horribly dirty bottles.

(*To be continued.*)

## Figures and Tables

**Figure f1:**